# How do we avoid disaster when exiting the European Medicines Agency? Making the most of Brexit in pharmaceutical regulation

**DOI:** 10.3332/ecancer.2017.ed67

**Published:** 2017-06-27

**Authors:** Anthony James Hatswell

**Affiliations:** BresMed Health Solutions, North Church House, 84 Queen Street, Sheffield S1 2DW, UK and; Department of Statistical Science, University College London, UK

**Keywords:** EMA, FDA, regulatory, market access

## Abstract

As the UK prepares to leave the EU, it must decide what path it is to take with a large number of regulatory and technical agencies who provide collaboration at the European level. In the case of pharmaceuticals, the European Medicines Agency (EMA) provides pan-European licencing for novel pharmaceuticals.

Should the UK depart from the EMA system, this article highlights the loss to patients immediately (slower access to novel treatments), and in the long term by having no access to some novel products as companies choose not to launch in the UK. The lack of access then may also preclude the access to these treatments as generic medicines, causing harm far into the future. The other costs considered are the cost for duplicating the functions of the EMA, or the alternative of using the decisions of other regulators without input to decisions made.

An alternative is then set out, of how the UK can prosper under ‘Brexit’, by remaining a member of the EMA, but accepting the decisions without political oversight (as currently happens with the European Commission). Additional freedom could be given to a UK regulator to accept decisions (where appropriate) from other agencies such as the Food & Drug Administration—further speeding access and making the UK a more attractive market.

Such an arrangement would put the UK in a better position than the good position it is in currently. This would give patients (both now and in the future) the best access to treatment possible, and promote/attract an industry which employs (directly and indirectly) 500,000 jobs.

## Introduction

Whilst a member of the European Union (EU), the United Kingdom (UK) has also been a part of various legal agencies set up to groups, from EurAtom (the atomic energy community) right through to the European Food Safety Authority (EFSA). Outside of their niche specialisms, these agencies are not well known and quietly go about their business. In the area of medicines, the relevant body is the European Medicines Agency (EMA), for now based in London.

The EMA has grown in importance, particularly since 1995 with the advent of the ‘Centralized procedure’ for drug approval, by which a single drug application, can grant marketing authorisation Europe wide [1]. This process is mandatory for most novel pharmaceuticals, and greatly reduces the overhead to companies, and ultimately the time to patients receiving treatment. Whilst not perfect or free of criticism [2], the ability to have a license in all member states from a single regulatory application is a great advantage.

All this however, may soon change—with the activation of Article 50 the UK will leave the EU. Given that the EMA must be based in an EU country, this in turn means the EMA must relocate [3]. More disruptive could be the shattering of the relationship—as of 30 March 2019, the UK will no longer be a part of the regulatory regime unless a deal is reached [4]—the content of such a deal is the more thorny issue—how should the UK regulate medicines? The issues involved are tremendously complex as the industry is necessarily formed of large multinational corporations.

The model used by the EMA is that of collaboration between national regulators, with a centralised staff. For each submission made for a new drug, a ‘rapporteur’ is appointed to assess the medicine—this role is filled by one of the national regulatory bodies that make up the agency who will perform highly technical reviews of not just the efficacy of the product, but the entire data package including areas such as pharmacokinetics and adverse events. The UK’s Medicines & Healthcare products Regulatory Agency (MHRA) performs this role about a third of the time—in the event of a complete schism there will be substantial gaps to fill for both agencies.

## How leaving the EMA would hurt the taxpayer, be bad for patients now, and disastrous for patients in the future

Few would argue there is no need for regulation in the pharmaceutical sector, thus we should consider the worst case of the UK if exiting the EMA entirely with no collaboration. Were this the case, the UK would face two choices: either duplicating the functions of the EMA entirely, or adopting their decisions (or those of another regulator). In the case of ‘rubber stamping’ the UK would have no say on decisions made, but need to adopt them regardless—for many reasons not a position that is desirable or likely.

Were the MHRA to duplicate functions, the MHRA would need to substantially expand to take on everything from licensing new medicines, to reviewing manufacturing in places such as India, and monitoring the safety of existing medicines. Duplication would substantially increase costs to companies, and to the MHRA (which in turn would need additional taxation, or user fees that would feed through to increased prices for medicines). Whilst in some cases a different outcome may be reached to what the EMA would conclude (for example on the risk/benefit of a new product), in most cases the duplication would be a dead loss to society.

Beyond the immediate financial costs, the harms to UK patients are twofold—firstly in delayed access. It has been shown in multiple studies that pharmaceuticals launch in the US before the EU [5, 6]. Given the US is a larger market in terms of patients and volume, this can be justified ethically and commercially. Other research then shows a delay from the EMA to Canada, Australia, and Switzerland [7, 8]. Whilst the EMA currently represents approximately a quarter of global pharmaceutical sales, the UK sits at 3% of the global market [9], realistically this would mean the UK experiencing a further delay before novel treatments are launched—larger markets like Japan would take precedence (with the limiting factor being staff time to perform analysis within companies). The claim of UK patients having to wait for 2–3 years for access to novel treatments does not sound unreasonable [10].

Going further there is the second form of harm to UK patients: no access whatsoever. With additional regulatory hurdles to sell pharmaceuticals in the UK, some drugs simply won’t get launched. In many cases this will be because the commercial calculation does not make sense for the UK alone. Either because the cost-effectiveness framework used by NICE often concludes that clinically effective medicines are not cost-effective [11], because incurring the cost of a regulatory review similar to that of the EMA for a single market may not be appealing, or due to limited company resources being focused on larger markets.

The impact of pharmaceuticals not launching would deprive patients of expensive (but effective) medicines in the medium term, but would have a catastrophic follow on impact for decades. The high priced medicines of today become generic, and often then become the backbone of cheap and effective therapy for the majority of patients. Today’s pertuzumab (Perjeta®, Roche Products Limited) is tomorrow’s paclitaxel. Without the original license, the UK would get neither. Alternatively once a medicine is generic elsewhere, companies may exploit the system to launch a product as ‘new’ with a substantial profit margin—as has been seen recently in the US with a decades old steroid, deflazacort [12].

## ‘Have cake and eat it’—a proposal for improving the regulation of pharmaceuticals in a UK outside of the EU

Whilst imperfect, the EMA represents a collaborative effort to provide stability in standards and regulations to an industry with a notoriously long development cycle. In addition to the benefits to the industry in helping to bring products to market, it benefits countries by spreading the cost and burden of essential services (such as monitoring of manufacturing). Close collaboration is in the interests of both the UK and EU in this area, and to completely exit most aspects of this framework would be nothing short of madness.

In terms of the regulation of novel pharmaceuticals, exiting the EMA mechanism entirely would harm patients in both the short and long term, and thus needs to be avoided. The ideal mechanism would therefore be the MHRA participating fully in the EMA process as at present—having input to decisions, and ensuring UK patients are not disadvantaged. There are however areas where the system can be improved, and a UK outside the EU would have the freedom to implement such changes. One major area for improvement is that once a scientific recommendation has been made by the EMA, it then goes to the European Commission (a political body with little scientific knowledge) for approval. This causes a delay in the approval of pharmaceuticals of just over two months, with no identifiable benefit whatsoever. A more liberal regulatory regime could bypass this step, adopting EMA decisions immediately (or following rapid approval from the appropriate minister/MHRA).

Going further, although there are differences in the attitudes of regulators, on the whole decisions made are extremely similar [13, 14]. Whilst the UK would not necessarily want to adopt decisions from other regulators as a matter of course, giving the MHRA (on behalf of patients) the freedom to adopt decisions made by other regulators such as the US Food and Drug Administration (if appropriate) would speed access to medicines, and keep the UK at the forefront of medical technology. Rather than falling behind the EU (as would happen with a complete exit), close collaboration with additional powers for the MHRA to act in the interest of patents in the UK could lead to a more advantageous system. How additional freedom can benefit the MHRA can be seen with the Early Access to Medicines scheme, where breakthrough medicines can be made available to patients whilst regulatory activities are completing [15].

## Conclusion

Whatever your political view on Brexit, we are approaching a time of great change. If the vital area of pharmaceutical regulation is neglected, irreparable harm could be done to the health of the UK population for generations to come. With careful planning and collaboration the opposite can occur—the UK can maintain or even advance its position as a leader in life sciences. Not only do patients require the latter, the life sciences sector is a large source of well-paid employment, and a large net exporter for the UK—the gross value added of workers in the sector being estimated to be twice the UK average [16] over the approximately 500,000 direct and indirect employees.

What is needed to avoid disaster, is for the case for collaboration to be made clearly by all stakeholders. Rather than treating the topic as a chip in trade negotiations, politicians need to give freedom to and empower the highly skilled staff at the MHRA and EMA to work together to find the best solution. Ideology should not trump the benefits of cooperation, beneficial to both parties, and whilst it will be sad to see the EMA leave the UK, this should not be the end of a productive relationship that has done great things for patients across Europe. If the situation is handled with the necessary respect and delicacy, it can continue to do so.

## Conflicts of interest

The author has no competing interests and has received no funding. The content of this article is the view of the author alone, and does not necessarily reflect any official position of BresMed or University College London.
